# Supplementary material for: Transcranial focused ultrasound-mediated neurochemical and functional connectivity changes in deep cortical regions in humans
Source: Nat Commun. 2023 Sep 1;14:5318. doi: 10.1038/s41467-023-40998-0 (PMC10474159; doi:10.1038/s41467-023-40998-0)
Supplement: Supplementary file 1 — Supplementary Information [file 41467_2023_40998_MOESM1_ESM.pdf]

Transcranial focused ultrasound-mediated neurochemical and functional connectivity changes in deep cortical regions in humans

Supplementary Tables

Supplementary Table 1. Neuronavigation parameters used and output of acoustic simulations of the left dorsal anterior cingulate cortex (dACC) target. 2
Supplementary Table 2. Neuronavigation parameters used and output of acoustic simulations of the left posterior cingulate cortex (PCC) target. 3
Supplementary Table 3. List of excluded medications. 8

Supplementary Figures

Supplementary Figure 1. Seed-based functional connectivity of the left dACC and left PCC during sham sessions. .. 4
Supplementary Figure 2. Increased functional connectivity with the dACC after PCC TUS. 5
Supplementary Figure 3. Magnetic resonance spectroscopy example acquisition. 6
Supplementary Figure 4. Regions showing increased functional connectivity after TUS compared with sham. 7

Supplementary Table 1. Neuronavigation parameters used and output of acoustic simulations of the left dorsal anterior cingulate cortex (dACC) target.

	Neuronavigation parameters			Simulated output										
ID	Transducer base coordinates	Target depth [mm]	Target coordinates	PPW	CFL	Maximum pressure coordinates	Focal depth [mm]	Maximum pressure [MPa]	MI	I_{SPPA} [W/cm^2]	I_{SPTA} [mW/cm^2]	Focal volume [mm^3]	Volume overlapping MRS voxel [mm^3]	Distance to COG of MRS voxel [mm]
TS01	59,147,230	63	78,129,163	6	0.2	80,131,166	61	0.674	0.96	15.42	1541.61	581	378	5.75
TS02	89,192,261	60	98,160,201	6	0.2	100,162,204	58	0.659	0.94	14.83	1483.34	499	354	2.15
TS03	71,187,219	61	82,160,155	6	0.2	84,161,159	59	0.690	0.98	16.08	1607.69	455	346	3.51
TS04	64,209,207	59	82,158,167	6	0.2	84,161,170	56	0.588	0.84	11.65	1165.00	620	445	7.14
TS05	61,202,214	66	83,162,155	6	0.2	85,165,158	64	0.652	0.92	14.25	1425.34	725	390	11.39
TS06	36,128,235	68	83,125,175	6	0.2	83,126,179	65	0.605	0.87	12.50	1250.32	809	525	5.20
TS07	67,182,240	62	98,173,177	6	0.2	98,175,182	58	0.669	0.97	15.58	1558.36	456	365	5.81
TS08	90,144,236	60	93,137,167	6	0.2	94,139,171	57	0.687	1.00	16.83	1682.69	457	298	8.91
TS09	50,159,233	62	80,143,169	6	0.2	81,146,174	60	0.643	0.91	13.88	1388.46	634	470	2.68
TS10	47,192,219	67	81,155,164	6	0.2	85,156,164	69	0.540	0.77	9.84	983.62	1146	518	12.20
TS11	70,161,234	60	83,146,168	6	0.2	85,148,172	57	0.716	1.02	17.36	1735.68	419	291	5.63
TS12	88,179,267	62	98,157,200	6	0.2	100,159,204	59	0.739	1.05	18.29	1828.76	470	338	3.99
TS13	73,162,232	55	80,140,170	6	0.2	82,143,175	52	0.683	0.97	15.73	1573.08	395	289	3.27
TS14	102,160,268	58	99,152,201	6	0.2	101,154,205	54	0.689	1.00	16.71	1670.84	419	293	4.26
TS16	77,152,256	55	85,139,193	6	0.2	87,141,194	54	0.697	1.01	16.89	1688.83	323	272	3.05
TS17	57,150,242	59	84,141,181	6	0.2	86,143,181	59	0.594	0.85	11.90	1190.41	658	345	9.20
TS18	66,127,255	65	85,125,187	6	0.2	88,126,184	66	0.644	0.92	14.21	1420.55	721	352	9.97
TS19	50,168,245	63	83,144,185	6	0.2	85,146,187	63	0.627	0.89	13.35	1334.74	724	452	6.22
TS20	62,172,228	54	84,147,173	6	0.2	86,149,177	52	0.563	0.81	10.88	1087.74	556	399	1.91
TS21	69,150,244	60	83,140,177	6	0.2	84,141,180	58	0.661	0.96	15.22	1522.27	462	350	2.67
TS23	59,166,219	55	83,147,160	6	0.2	84,149,166	52	0.641	0.92	14.04	1403.51	356	301	2.95
TS24	73,158,229	52	85,145,169	6	0.2	86,147,172	50	0.724	1.05	18.22	1822.03	270	239	2.53
TS25	77,156,218	60	83,147,150	6	0.2	84,149,155	56	0.659	0.95	14.95	1494.89	470	306	6.33
TS26	70,177,222	56	83,145,165	6	0.2	85,147,170	53	0.670	0.95	15.00	1499.75	402	322	2.79

ID: subject identifier; PPW: points-per-wavelength; CFL: Courant–Friedrichs–Lewy condition number; MI: mechanical index; I_{SPPA}: spatial-peak pulse-averaged intensity; I_{SPTA}: spatial-peak temporal-averaged intensity; MRS: magnetic resonance spectroscopy; COG: centre of gravity.

Supplementary Table 2. Neuronavigation parameters used and output of acoustic simulations of the left posterior cingulate cortex (PCC) target.

	Neuronavigation parameters			Simulated output										
**ID**	**Transducer base coordinates**	**Target depth [mm]**	**Target coordinates**	**PPW**	**CFL**	**Maximum pressure coordinates**	**Focal depth [mm]**	**Maximum pressure [MPa]**	**MI**	**ISPPA [W/cm²]**	**ISPTA [mW/cm²]**	**Focal volume [mm³]**	**Volume overlapping MRS voxel [mm³]**	**Distance to COG of MRS voxel [mm]**
TS01	66,3,201	68	79,67,159	6	0.2	78,64,161	66	0.637	0.90	13.59	1359.10	723	457	4.87
TS02	106,57,262	75	98,100,194	6	0.2	98,100,196	73	0.654	0.93	14.38	1437.62	942	479	7.54
TS03	75,111,231	76	82,104,150	6	0.2	81,106,153	72	0.709	1.01	17.10	1710.43	723	407	10.19
TS04	41,67,222	72	83,94,160	6	0.2	82,94,162	71	0.671	0.96	15.40	1539.81	652	486	2.62
TS05	74,83,244	70	81,105,170	6	0.2	81,108,166	74	0.594	0.84	11.74	1173.89	925	335	11.22
TS06	60,21,215	69	82,71,160	6	0.2	81,72,161	69	0.649	0.93	14.44	1444.45	728	453	5.68
TS07	89,79,234	77	99,113,160	6	0.2	99,112,162	74	0.665	0.95	14.90	1489.76	891	467	7.95
TS08	60,46,215	72	83,90,153	6	0.2	84,94,150	77	0.591	0.84	11.82	1181.84	1031	346	15.87
TS09	91,42,220	73	84,89,155	6	0.2	84,89,156	73	0.606	0.87	12.58	1258.14	982	464	6.20
TS10	80,118,255	82	84,107,174	6	0.2	86,109,171	82	0.647	0.92	14.21	1420.71	845	514	6.71
TS11	87,52,213	62	84,97,158	6	0.2	84,97,159	61	0.675	0.96	15.23	1523.36	550	344	8.42
TS12	93,49,254	64	98,94,197	6	0.2	98,95,195	66	0.667	0.96	15.27	1526.88	609	356	6.84
TS13	68,50,237	69	79,81,167	6	0.2	79,84,163	74	0.640	0.91	13.90	1390.48	922	577	8.38
TS14	104,64,256	64	99,100,190	6	0.2	99,99,193	63	0.682	0.97	15.83	1583.13	589	387	5.65
TS16	50,51,225	71	82,82,171	6	0.2	83,84,167	67	0.738	1.06	18.78	1878.20	509	218	12.12
TS17	77,52,244	68	83,85,175	6	0.2	83,87,174	70	0.613	0.87	12.53	1253.25	833	437	8.32
TS18	72,9,207	76	85,78,164	6	0.2	84,79,165	77	0.659	0.94	14.64	1463.63	901	495	9.79
TS19	30,51,226	76	84,83,174	6	0.2	86,86,171	80	0.610	0.86	12.41	1240.69	1025	557	6.86
TS20	69,60,235	63	82,90,171	6	0.2	82,91,172	62	0.615	0.88	12.97	1297.19	633	390	5.30
TS21	65,34,218	68	82,82,162	6	0.2	83,84,162	69	0.600	0.85	12.11	1210.50	842	487	3.57
TS23	65,67,219	56	83,92,160	6	0.2	83,93,161	56	0.687	0.98	15.85	1585.43	384	311	3.65
TS24	86,29,196	64	84,87,151	6	0.2	84,84,154	60	0.695	0.99	16.25	1624.72	505	369	5.00
TS25	77,47,198	63	84,92,141	6	0.2	84,92,142	63	0.680	0.98	15.99	1599.28	493	345	3.49
TS26	66,58,227	70	84,91,159	6	0.2	83,91,161	68	0.673	0.96	15.32	1531.89	648	418	3.96

ID: subject identifier; PPW: points-per-wavelength; CFL: Courant–Friedrichs–Lewy condition number; MI: mechanical index; I_{SPPA}: spatial-peak pulse-averaged intensity; I_{SPTA}: spatial-peak temporal-averaged intensity; MRS: magnetic resonance spectroscopy; COG: centre of gravity.

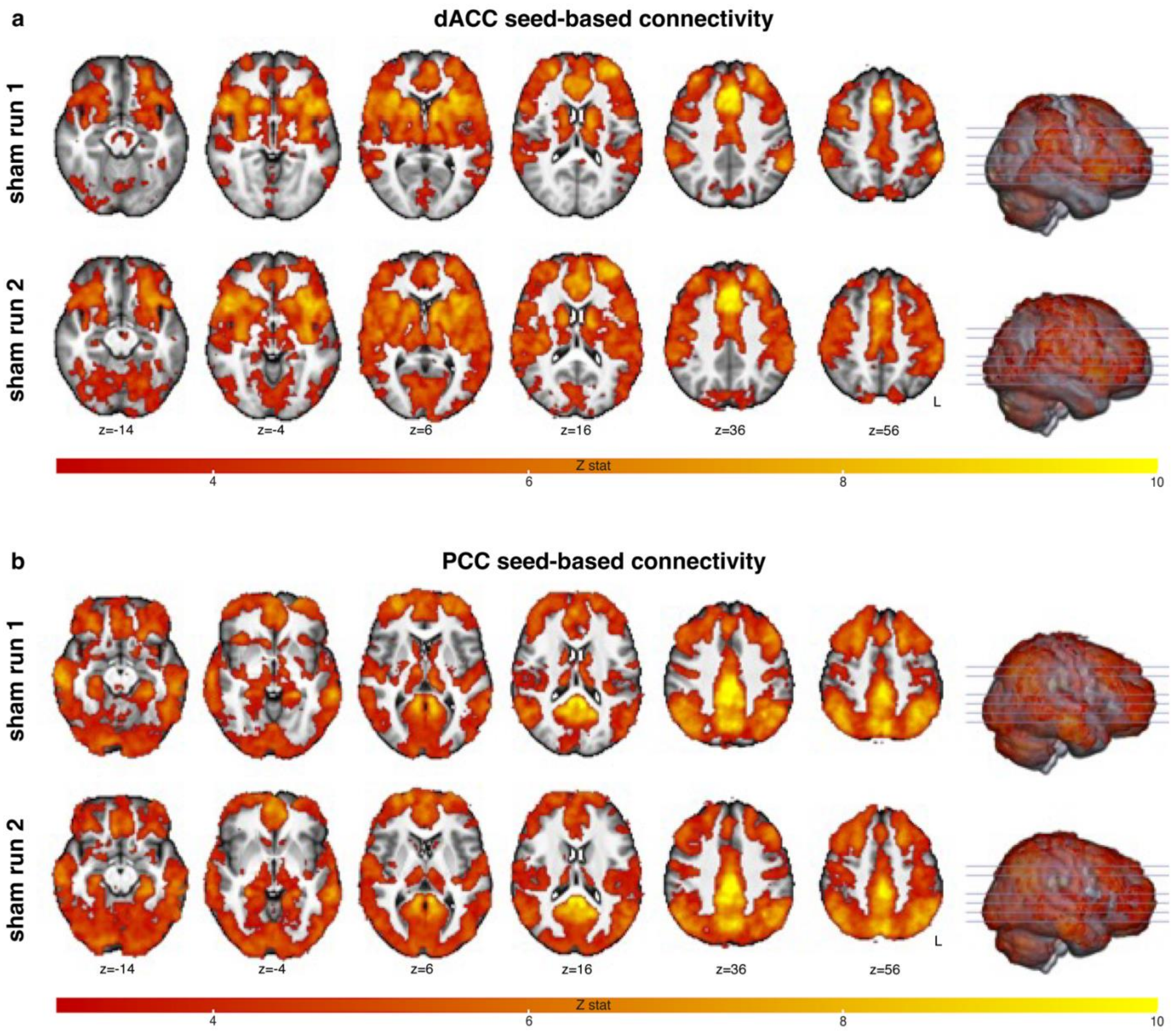

Supplementary Figure 1. Seed-based functional connectivity during sham sessions. a Seed-based functional connectivity of the left dorsal anterior cingulate cortex (dACC) during the sham transcranial ultrasound stimulation (TUS) sessions. **b** Seed-based functional connectivity left posterior cingulate cortex (PCC) during the sham TUS sessions. Sham run 1 denotes the early rsfMRI run at approximately 13 minutes after sham TUS was applied. Sham Run 2 denotes the late rsfMRI run at approximately 46 minutes after sham TUS. Whole-brain maps are overlaid on the average T1-weighted MRI of all participants. There were no significant differences between the early and late rsfMRI runs during the sham session for either the left dACC or the left PCC seed.

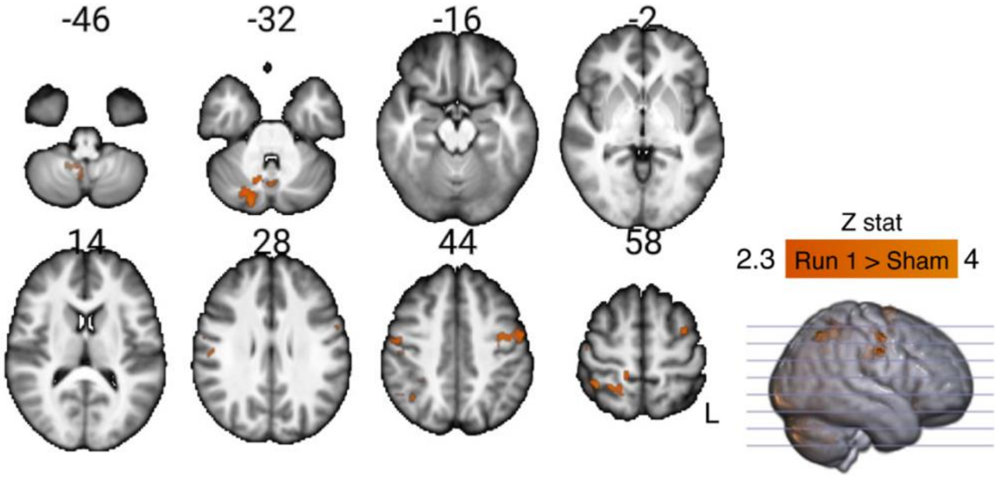

Supplementary Figure 2. Increased functional connectivity with the dorsal anterior cingulate cortex (dACC) after transcranial ultrasound stimulation (TUS) applied to the posterior cingulate cortex (PCC). Functional connectivity changes with the dACC seed after TUS applied to the PCC during the early rsfMRI run (i.e., approximately 13 minutes post-TUS) compared with the average of the two rsfMRI runs during the sham session. Whole-brain Z-statistic images (one-sided contrast, thresholded using clusters determined by Z > 2.3 and a FWER corrected cluster significance threshold of p = 0.05) illustrate regions showing increased functional connectivity based on a mass-univariate whole-brain GLM.

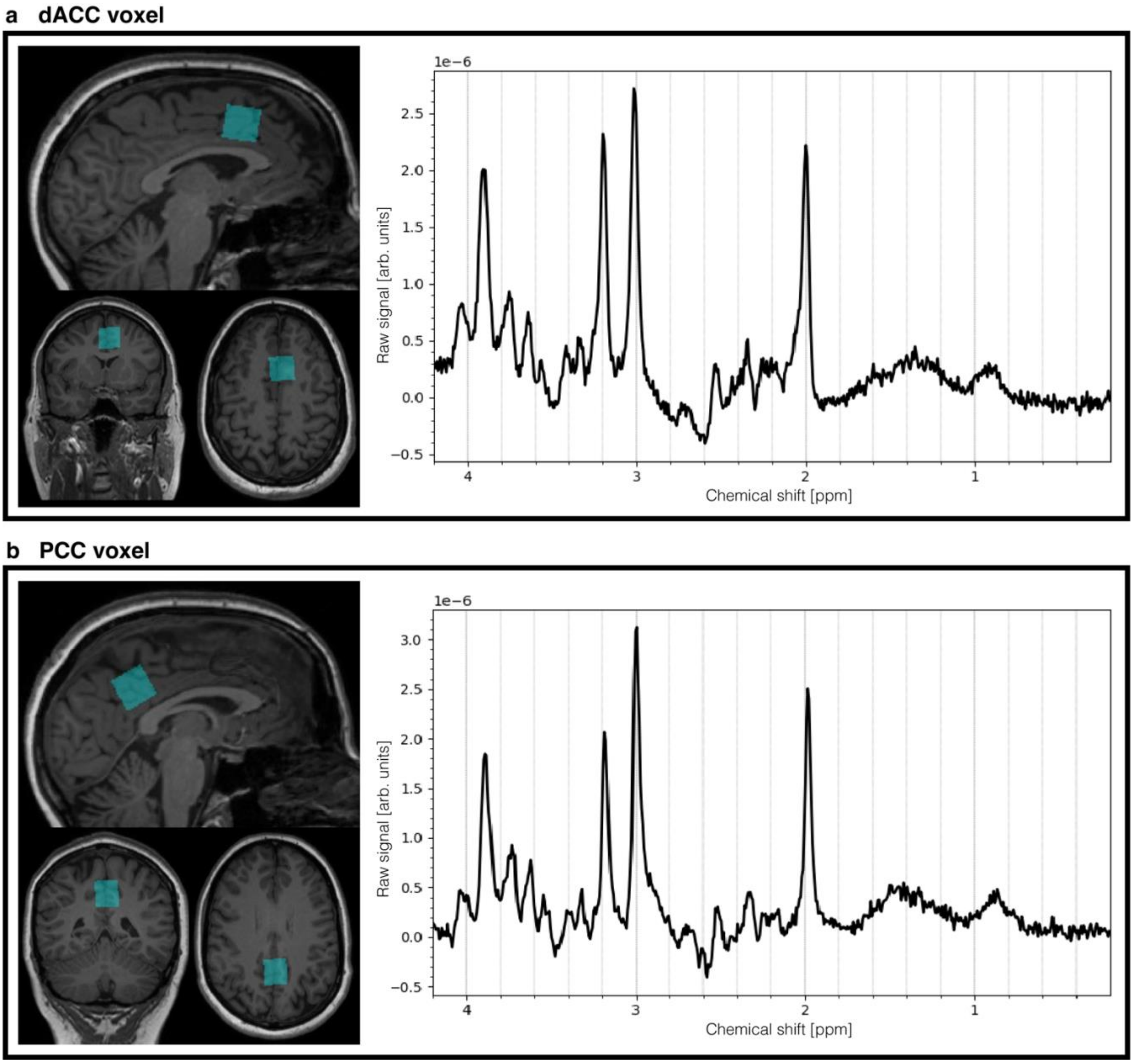

Supplementary Figure 3. Magnetic resonance spectroscopy (MRS) example acquisition. Representative spectrum and voxel location (cyan squares) acquired from **a** the left dorsal anterior cingulate cortex (dACC) and **b** the left posterior cingulate cortex (PCC) MRS voxel in one individual.

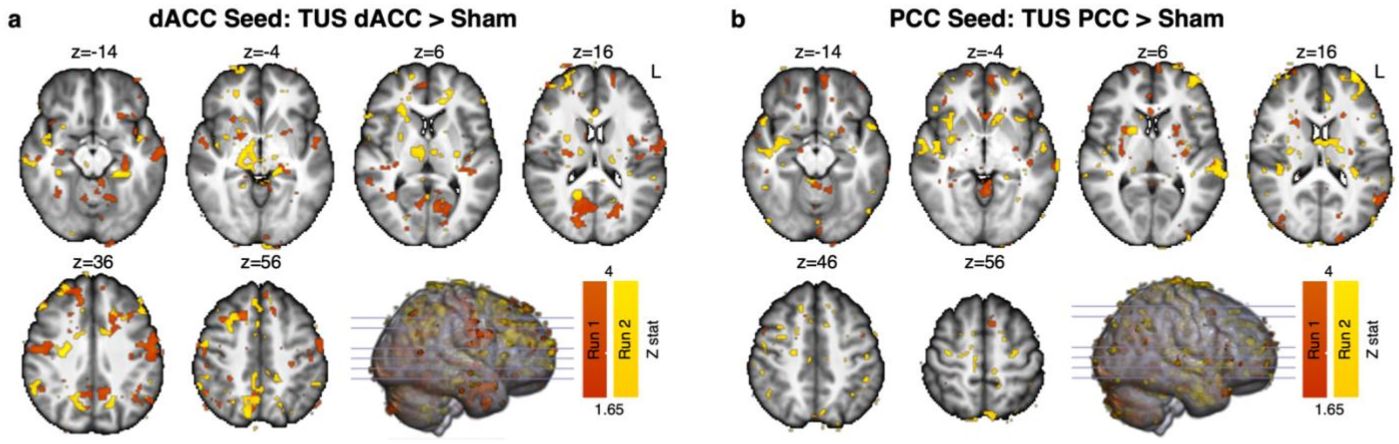

Supplementary Figure 4. Regions showing increased functional connectivity after transcranial ultrasound stimulation (TUS) compared with sham. Regions showing increased functional connectivity with **a** the dorsal anterior cingulate cortex (dACC) seed after TUS was applied to the dACC, and **b** the posterior cingulate cortex (PCC) seed after TUS was applied to the PCC, compared with sham. Clusters in orange represent regions with significantly higher functional connectivity at approximately 13 minutes after TUS (i.e., fMRI Run 1), and clusters in yellow show regions with significantly higher functional connectivity at approximately 46 minutes after TUS (fMRI Run 2) compared with the corresponding sham runs. Statistical maps here are shown at $Z > 1.65$ uncorrected, to show a similar spatial extent to statistically significant results obtained when comparing active TUS conditions to the average of both runs in the sham condition (Figure 5 of the main manuscript).

Supplementary Table 3. List of excluded medications. Participants who are currently taking or have recently withdrawn from the below drugs are excluded from participating in the study. This list was adapted from safety considerations for repetitive transcranial magnetic stimulation (rTMS) research studies.

Category 1: Drugs that present a **strong potential hazard** for application of rTMS

Imipramine
Amitriptyline
Doxepine
Nortriptyline
Maprotiline
Chlorpromazine
Clozapine
Foscarnet
Ganciclovir
Ritonavir
Amphetamines
Cocaine
MDMA
Ecstasy
Phencyclidine (PCP)
Ketamine
Gamma-hydroxybutyrate (GHB)
Theophylline

Category 2: Drugs that present a **relative hazard** for application of rTMS

Mianserin	Ampicillin
Fluoxetine	Cephalosporins
Fluvoxamine	Metronidazole
Paroxetine	Isoniazid
Sertraline	Levofloxacin
Citalopram	Cyclosporine
Reboxetine	Chlorambucil
Venlafaxine	Vincristine
Duloxetine	Methotrexate
Bupropion	Cytosine arabinoside
Mirtazapine	Carmustine (BCNU)
fluphenazine	Lithium
Pimozide	Anticholinergics
Haloperidol	Antihistamines
Olanzapine	Risperidone
Quetiapine	Chloroquine
Aripiprazole	Mefloquine
Ziprasidone	Imipenem
Penicillin	Sympathomimetics

Category 3: Drugs that pose a withdrawal risk

Barbiturates
Benzodiazepines
Meprobamate
Chloral hydrate
