# Supplementary material for: Transcranial focused ultrasound-mediated neurochemical and functional connectivity changes in deep cortical regions in humans
Source: Nat Commun. 2023 Sep 1;14:5318. doi: 10.1038/s41467-023-40998-0 (PMC10474159; doi:10.1038/s41467-023-40998-0)
Supplement: Supplementary file 3 — Reporting Summary [file 41467_2023_40998_MOESM3_ESM.pdf]

Corresponding author(s): Dr Elsa Fouragnan

Last updated by author(s): Aug 2, 2023

Reporting Summary

Nature Portfolio wishes to improve the reproducibility of the work that we publish. This form provides structure for consistency and transparency in reporting. For further information on Nature Portfolio policies, see our Editorial Policies and the Editorial Policy Checklist.

Statistics

For all statistical analyses, confirm that the following items are present in the figure legend, table legend, main text, or Methods section.

n/a	Confirmed	
☐	☒	The exact sample size (n) for each experimental group/condition, given as a discrete number and unit of measurement
☐	☒	A statement on whether measurements were taken from distinct samples or whether the same sample was measured repeatedly
☐	☒	The statistical test(s) used AND whether they are one- or two-sided<br>*Only common tests should be described solely by name; describe more complex techniques in the Methods section.*
☐	☒	A description of all covariates tested
☐	☒	A description of any assumptions or corrections, such as tests of normality and adjustment for multiple comparisons
☐	☒	A full description of the statistical parameters including central tendency (e.g. means) or other basic estimates (e.g. regression coefficient) AND variation (e.g. standard deviation) or associated estimates of uncertainty (e.g. confidence intervals)
☐	☒	For null hypothesis testing, the test statistic (e.g. F, t, r) with confidence intervals, effect sizes, degrees of freedom and P value noted<br>*Give P values as exact values whenever suitable.*
☒	☐	For Bayesian analysis, information on the choice of priors and Markov chain Monte Carlo settings
☒	☐	For hierarchical and complex designs, identification of the appropriate level for tests and full reporting of outcomes
☐	☒	Estimates of effect sizes (e.g. Cohen's d, Pearson's r), indicating how they were calculated

Our web collection on statistics for biologists contains articles on many of the points above.

Software and code

Policy information about availability of computer code

Data collection	Transcranial ultrasound stimulation was delivered using the NeuroFUS TPO and CTX-500-4 transducer (Brainbox Ltd., Cardiff, UK). Neuronavigation was performed with the Brainsight software v 2.4.11 (Rogue Research Inc., Montréal, Québec, Canada).<br>MRI scans were acquired on a Siemens MAGNETOM Prisma 3T scanner (VE11E, Siemens Healthineers, Erlangen, Germany) with a 32-channel head coil using sequences from the University of Minnesota Centre for Magnetic Resonance Research (CMRR; multiband fMRI: https://www.cmrr.umn.edu/multiband and single-voxel spectroscopy MEGA-PRESS: https://www.cmrr.umn.edu/spectro).
Data analysis	Acoustic simulations were performed using the k-Wave Toolbox (version 1.4) implemented in MATLAB (R2020b, MathWorks, Inc.). Code for generating pseudo-CT from T1-weighted MR images and for running acoustic simulations as described in this work are available on GitHub: https://github.com/sitiny/mr-to-pct and https://github.com/sitiny/BRIC_TUS_Simulation_Tools.<br>MRS processing and analysis was performed in MATLAB (R2020b, MathWorks, Inc.) using Gannet (version 3.1; http://www.gabamrs.com/) and SPM12 for segmentation (Ashburner & Friston, 2005). Statistical analyses were performed in JAMOVI version 2.0.0 (https://www.jamovi.org/).<br>FMRI data were pre-processed and analysed using FEAT (FMRI Expert Analysis Tool) and MELODIC tools from the FMRIB Software Library v6.0 (FSL; www.fmrib.ox.ac.uk/fsl).

For manuscripts utilizing custom algorithms or software that are central to the research but not yet described in published literature, software must be made available to editors and reviewers. We strongly encourage code deposition in a community repository (e.g. GitHub). See the Nature Portfolio guidelines for submitting code & software for further information.

Data

Policy information about availability of data

All manuscripts must include a data availability statement. This statement should provide the following information, where applicable:

- Accession codes, unique identifiers, or web links for publicly available datasets
- A description of any restrictions on data availability
- For clinical datasets or third party data, please ensure that the statement adheres to our policy

The raw and processed MR data and acoustic simulation data generated in this study have been deposited in the Open Science Framework database under the CC-By Attribution 4.0 License: https://osf.io/rp5g4/. Human Protein Atlas data was accessed via the online portal: https://www.proteinatlas.org/humanproteome/brain/cerebral+cortex. Source data are provided with this paper.

Human research participants

Policy information about studies involving human research participants and Sex and Gender in Research.

Reporting on sex and gender	Sex was determined based on participant self-reports. In the study design, we aimed to recruit a balanced number of participants from both sexes (14 females and 10 males included in the study). No information on gender was collected. No sex-based analyses were performed as the measured parameters are not known to be influenced by sex.
Population characteristics	Twenty-four healthy volunteers (14 female) aged between 22 and 53 years (mean = 33.8, s.d. 404 ± 9.7) participated in the study. Participants reported no current diagnosis of neurological or psychiatric disorders and were not taking any medications known to affect brain excitability at the time of the study.
Recruitment	Participants were recruited through advertisements posted via the University's email circulation lists, on the lab website (www.elsa-fouragnan.com), on social media (Twitter) and through word-of-mouth. Participants were compensated £30 for completing each session and travel expenses up to £10 per session.<br>We screened all individuals who expressed interest in participating in the study and those who met the inclusion criteria were recruited. Potential sources of bias could be present in that individuals who were interested in research or who were acquainted with the researchers may be more likely to self-select and volunteer for the study. This may bias the sample to those within the University academic community, and thus the results could reflect how the brains of academics respond to neurostimulation. However, this potential bias in the sample is mitigated by the fact that our sample included 11 participants (46%) who were not from the University academic community.
Ethics oversight	The study was approved by the University of Plymouth Faculty of Health Staff Research Ethics and Integrity Committee (reference ID: 2487; date: 13/12/2021). Written informed consent was obtained from all participants after experimental procedures were explained in full.

Note that full information on the approval of the study protocol must also be provided in the manuscript.

Field-specific reporting

Please select the one below that is the best fit for your research. If you are not sure, read the appropriate sections before making your selection.

[x] Life sciences   [ ] Behavioural & social sciences   [ ] Ecological, evolutionary & environmental sciences

For a reference copy of the document with all sections, see nature.com/documents/nr-reporting-summary-flat.pdf

Life sciences study design

All studies must disclose on these points even when the disclosure is negative.

Sample size	Since there are no previous studies of TUS effects MR spectroscopy, we based our sample size estimate on effect sizes from studies of transcranial direct current stimulation with MRS (Cohen's d approx. 1.4; n = 16 and n = 12; Stagg et al., 2009, Bachtiar et al., 2015), from a study of theta-burst TUS on motor evoked potential amplitude changes (Cohen's d approx. 0.8; n = 15; Zeng et al. 2021) and from a study using a different offline TUS protocol on fMRI connectivity changes (Cohen's d approx. 3.6; n = 9; from Sanguinetti et al., 2020). We chose the most conservative effect size of 0.8 for our power calculation.<br>We used G*Power (https://www.psychologie.hhu.de/arbeitsgruppen/allgemeine-psychologie-und-arbeitspsychologie/gpower) to conduct a power analysis to determine the sample size. Our goal was to obtain 0.95 power to detect an effect size of 0.8 at the standard 0.05 alpha error probability level. Our power analysis revealed that for a minimum effect size of 0.8, a sample size of 19 is required. These estimates broadly accord with our original predictions but illustrate that we may be close to the limits of detection. In view of these calculations, we have increased the target sample size to a maximum of 25 participants.
Data exclusions	Exclusion criteria were established before any analyses were performed. MRS spectra were visually inspected for spectral artifacts, including lipid contamination, subtraction errors and a non-constant baseline. One data point (out of 144 data points in total: 3 sessions x 2 voxels x 24 participants) was excluded based on this. Outliers on the following quality metrics were also excluded: FWHM, GABA+ signal-to-noise ratio

	(SNR), linewidth, and model fit errors. Ten data points were excluded based on these metrics. fMRI data were excluded if motion exceeded 0.25 mm (relative displacement between consecutive volumes) for more than 50% of the volumes, or if image artefacts were discovered during visual data quality inspection. No data were excluded on this basis.
Replication	We did not replicate any experiments in individuals. Each individual underwent the same study procedure and set of MRI scans, with only the TUS condition changed - each participant had one TUS of dACC, one TUS of PCC and one sham session.
Randomization	We used block randomisation to assign each participant to one of six "blocks" that determine the order of the TUS sessions and sham: 1) sham, dACC, PCC, 2) sham, PCC, dACC, 3) dACC, sham, PCC, 4) dACC, PCC, sham, 5) PCC, sham, dACC, or 6) PCC, dACC, sham. The random list used to create these six blocks was created using the List Randomizer at http://random.org.
Blinding	The experiment was designed as a single-blind study. Participants were blinded to the TUS study conditions. The experimenters and researchers who analysed the data were aware of the study conditions, which could possibly introduce some bias during the data collection and analysis. However this was unavoidable because the regions stimulated would have required placing the transducer on different parts of the head, and it was not possible to operate the TUS system in a blinded fashion, so experimenters would know whether stimulation was applied and to which location. In order to ensure participants were blinded to the study conditions, we designed the sound delivered via bone conduction headphones during the sham condition to closely match the TUS condition and turned off the TUS system so that no acoustic energy is delivered. All other procedures were kept the same across conditions.

Reporting for specific materials, systems and methods

We require information from authors about some types of materials, experimental systems and methods used in many studies. Here, indicate whether each material, system or method listed is relevant to your study. If you are not sure if a list item applies to your research, read the appropriate section before selecting a response.

Materials & experimental systems

n/a	Involved in the study
☒	☐ Antibodies
☒	☐ Eukaryotic cell lines
☒	☐ Palaeontology and archaeology
☒	☐ Animals and other organisms
☒	☐ Clinical data
☒	☐ Dual use research of concern

Methods

n/a	Involved in the study
☒	☐ ChIP-seq
☒	☐ Flow cytometry
☐	☒ MRI-based neuroimaging

Magnetic resonance imaging

Experimental design

Design type	Resting-state - between subject rsfMRI design.
Design specifications	Each participant underwent two 5-minute resting-state functional MRI scans per session. Before each scan, they were instructed to keep their eyes closed, relax and let their minds wander, but to try not to fall asleep.
Behavioral performance measures	There was no task during the resting-state scan.

Acquisition

Imaging type(s)	Structural, functional, and single-voxel spectroscopy
Field strength	3 Tesla
Sequence & imaging parameters	Structural: T1-weighted magnetisation-prepared rapid gradient echo (MPRAGE) sequence acquired in the sagittal plane for MRS voxel planning (2100 ms repetition time (TR), 475 2.26 ms echo time (TE), 900 ms inversion time, 8° flip angle (FA), GRAPPA acceleration factor of 2, 256 × 256 476 mm field of view, 176 slices and 1 mm3 isotropic voxels)<br>Functional: gradient echo echo planar imaging (GE-EPI) from the CMRR multiband fMRI sequences (acquisition plane approximately parallel to the AC-PC line, 2000 ms TR, 30 ms TE, 74° FA, 2.5 mm slice thickness, no slice gap, multi-band acceleration factor of 2, and 60 interleaved slices of 80 × 80 matrix size, giving a voxel size of 2.5 × 2.5 × 2.5 mm3)<br>Spectroscopy: 2 × 2 × 2 cm3 voxel, CMRR single-voxel spectroscopy MEGA-PRESS sequence, 2000 ms TR, 68 ms TE, with VAPOR water suppression, 128 averages, Edit On/Off frequency at 1.90/7.50, editing pulse bandwidth 50.55 Hz, number of spectral points 2048, spectral width 1850 Hz, water unsuppressed reference: 16 averages
Area of acquisition	A whole brain scan was used for structural and functional MRI acquisitions. MR spectroscopy was acquired in two pre-defined regions of interest, the dorsal anterior cingulate cortex (dACC) and the posterior cingulate cortex (PCC). The dACC and PCC targets were identified based on an initial co-registration with the Montreal Neurological Institute (MNI) coordinate space at x = -5, y = 24, z = 30 for the dACC and x = -5, y = -35, z = 35 for the PCC. This was then adjusted based on anatomical landmarks on each individual's T1-weighted MRI. The dACC target was aligned with the back of the genu and superior-most point of the body of the corpus callosum, centred on a patch of grey matter in the cingulate

gyrus. The PCC target was aligned with the middle of the splenium of the corpus callosum, roughly in line with the ascending ramus of the cingulate sulcus and centred on a patch of grey matter just anterior to this, below the cingulate sulcus. MRS was acquired in a voxel centred on the target from the TUS session to ensure overlap between the TUS focus and MRS acquisition.

Diffusion MRI ☐ Used ☒ Not used

Preprocessing

Preprocessing software	MRS preprocessing was performed with Gannet (v3.1; http://www.gabamrs.com/). We used the default preprocessing steps including 3 Hz line broadening, correction for frequency and phase errors by spectral registration, outlier rejection, time averaging, and eddy current correction.<br>fMRI preprocessing was performed using tools from the FMRIB Software Library v6.0 (FSL; www.fmrib.ox.ac.uk/fsl). Pre-processing included motion correction (MCFLIRT), B0 field inhomogeneity correction, brain extraction (BET), spatial smoothing (5 mm FWHM) and highpass filtering (0.01 Hz).
Normalization	fMRI data were normalised to the MNI standard space via a linear transform (FSL FLIRT) to each individual's high-resolution T1-weighted MRI and a non-linear transform to the MNI152 template (FSL FNIRT).
Normalization template	MNI152_T1_2mm_brain (as packaged with the FSL software)
Noise and artifact removal	Motion outliers were identified using the fslmotionoutliers tool and were included as nuisance covariates in the model along with the average signal from the white matter and cerebrospinal fluid, and the six motion parameters from the motion correction step.
Volume censoring	We did not remove volumes during which significant movement occurred, instead, we used our motion-related artifacts (i.e. regression of motion parameters) as regressors of non interest that were not convolved in our general linear models.

Statistical modeling & inference

Model type and settings	We employed a mass univariate approach within the general linear model framework to perform whole-brain statistical analyses of functional data as implemented in the FMRIB Software Library. To assess seed-based functional connectivity, the average timeseries was sampled from each seed (TUS focal maximum volume) and used as the variable of interest in a voxel-wise whole-brain GLM implemented using FSL's FEAT, with the nuisance regressors described above as variables of non-interest. Seed-based functional connectivity for each seed was first combined at the subject level, comparing each TUS session and run against the mean of the sham runs with a fixed effects model within each subject. Comparisons across subjects were done using a mixed-effects model (FLAME1+2) with automatic outlier detection with age and sex as covariates.
Effect(s) tested	The effect tested was a measure of whole-brain functional connectivity of the seed region of interest derived from the voxel-wise whole-brain general linear model described above.

Specify type of analysis: ☐ Whole brain ☐ ROI-based ☒ Both

Anatomical location(s)	The seeds for the seed-based analysis were the subject-specific TUS focal volumes obtained from the dACC and PCC acoustic simulations. To create the subject-specific TUS seed, a binary mask was first created from the top 25% maximum pressure intensities in the simulated pressure field. This binary volume was then dilated by two voxels to give an average seed volume of 805 ± 162 mm3 for the dACC and 1003 ± 178 mm3 for the PCC (for reference, a typical 6 mm radius spherical seed used in seed-based functional connectivity analyses has a volume of 905 mm3).
Statistic type for inference (See Eklund et al. 2016)	Whole-brain Z-statistic maps were thresholded using clusters determined by $Z > 2.3$ ($p = 0.05$) and a familywise error-corrected cluster significance threshold of $p = 0.05$.
Correction	FWE-corrected cluster significance threshold of $p = 0.05$

Models & analysis

n/a	Involved in the study
☐	☒ Functional and/or effective connectivity
☒	☐ Graph analysis
☒	☐ Multivariate modeling or predictive analysis

Functional and/or effective connectivity	The parameter estimate derived from the voxel-wise whole-brain general linear model was used as a measure of seed-based functional connectivity.
